# Rescue percutaneous transluminal septal myocardial ablation for difficult ventilator weaning in drug-resistant hypertrophic obstructive cardiomyopathy: a case report

**DOI:** 10.1093/ehjcr/ytaf435

**Published:** 2025-09-02

**Authors:** Keisuke Nakabayashi, Takeshi Hayashi, Shota Kaiga, Nobuhito Kaneko, Minoru Shimizu

**Affiliations:** Heart Center, Kasukabe Chuo General Hospital, 5-9-4 Midori-cho, Kasukabe, Saitama 344-0063, Japan; Heart Center, Kasukabe Chuo General Hospital, 5-9-4 Midori-cho, Kasukabe, Saitama 344-0063, Japan; Heart Center, Kasukabe Chuo General Hospital, 5-9-4 Midori-cho, Kasukabe, Saitama 344-0063, Japan; Heart Center, Kasukabe Chuo General Hospital, 5-9-4 Midori-cho, Kasukabe, Saitama 344-0063, Japan; Heart Center, Kasukabe Chuo General Hospital, 5-9-4 Midori-cho, Kasukabe, Saitama 344-0063, Japan

**Keywords:** Hypertrophic obstructive cardiomyopathy, Percutaneous transluminal septal ablation, Mechanical ventilator weaning, Volume optimization, Cardiopulmonary arrest, Case report

## Abstract

**Background:**

Hypertrophic obstructive cardiomyopathy is a genetic cardiac disorder characterized by left ventricular outflow tract (LVOT) obstruction that can lead to haemodynamic instability. Managing preload in mechanically ventilated patients with hypertrophic obstructive cardiomyopathy is challenging because volume depletion exacerbates LVOT obstruction, whereas fluid overload induces pulmonary congestion. Weaning from mechanical ventilation (MV) in such cases requires precise volume management; however, conventional pharmacological therapy may be insufficient. Moreover, the role of percutaneous transluminal septal myocardial ablation (PTSMA) in the acute phase of symptomatic hypertrophic obstructive cardiomyopathy remains unclear.

**Case Summary:**

A 51-year-old woman was admitted with cerebral haemorrhage and underwent emergency craniotomy. The patient experienced repeated episodes of acute heart failure, leading to several resuscitation attempts and requiring extended MV. Echocardiography revealed severe LVOT obstruction (peak LVOT pressure gradient, 119.7 mmHg). Attempts at volume optimization and pharmacological therapy failed to wean from MV or achieve haemodynamic stability. Given the patient’s critical condition, rescue PTSMA was performed, which significantly reduced the LVOT gradient, thereby allowing aggressive volume reduction with haemodialysis and successful extubation within a week.

**Discussion:**

This case highlights the role of PTSMA as a rescue therapy for patients with hypertrophic obstructive cardiomyopathy experiencing difficulty weaning from MV and haemodynamic instability. Further studies are warranted to evaluate the safety, efficacy, and long-term outcomes of PTSMA as an acute-phase intervention in critically ill patients with hypertrophic obstructive cardiomyopathy.

Learning points

- Ventilator weaning in patients with hypertrophic obstructive cardiomyopathy (HOCM) is challenging, as fluid-restrictive strategies may worsen left ventricular outflow tract obstruction, causing haemodynamic instability.
- Percutaneous transluminal septal myocardial ablation may be considered as a rescue option when medical therapy fails to relieve obstruction in ventilator-dependent patients with HOCM.
- Timely recognition and intervention are critical in HOCM-related extubation failure to avoid prolonged mechanical ventilation and its complications.

## Introduction

Hypertrophic obstructive cardiomyopathy (HOCM), a genetic cardiac disease characterized by left ventricular hypertrophy that induces left ventricular outflow tract (LVOT) obstruction, can lead to severe outcomes, including hypotension and pulmonary congestion due to elevated left ventricular end-diastolic pressure, depending on the preload of the left ventricle. In cases of HOCM with mechanical ventilation (MV), the therapeutic window for preload is narrow, making volume control challenging. Because positive-pressure ventilation limits venous return to the right heart, an adequate volume load is required to maintain left ventricular preload. Nevertheless, in patients with overt heart failure, excessive fluid administration can cause pulmonary congestion. During attempts to wean from MV, a dry fluid balance is preferred to avoid cardiovascular weaning failure.^1^ However, in patients with HOCM, this fluid strategy can aggravate LVOT obstruction, leading to haemodynamic instability, making ventilator weaning challenging.

Percutaneous transluminal septal myocardial ablation (PTSMA), also known as transcoronary ablation of septal hypertrophy (TASH),^2^ is a minimally invasive alternative to surgical myectomy; however, it is performed electively in patients with chronic symptoms.^3^ Reports describing urgent PTSMA in patients with symptomatic HOCM with haemodynamic instability and challenging ventilator weaning are limited.^4^ Herein, we present a patient who was initially admitted and intubated for a noncardiac condition but developed extubation failure due to HOCM. Successful extubation was achieved using PTSMA combined with subsequent volume management and haemodialysis, highlighting the potential of PTSMA in critically ill patients.

## Summary figure

**Figure:**
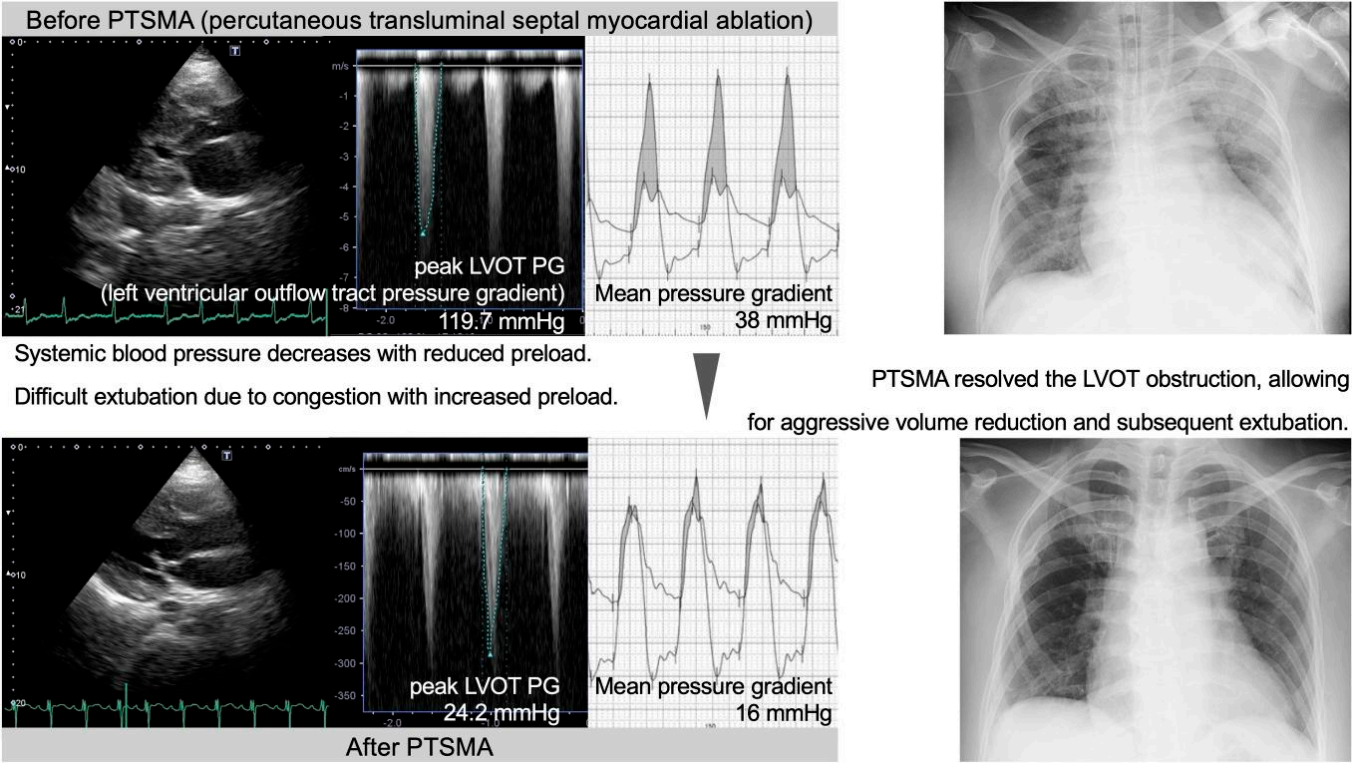


## Case presentation

**Figure 1:**
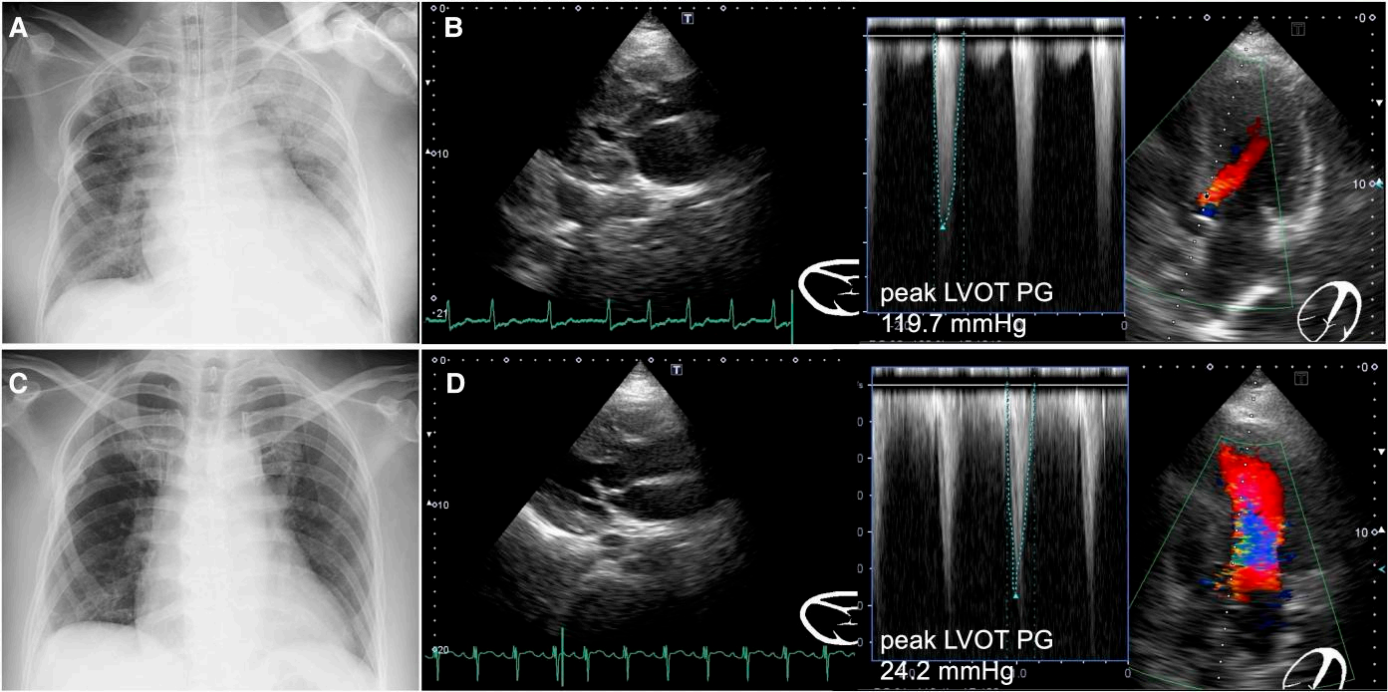
Chest X-ray and transthoracic echocardiography (TTE) before and after percutaneous transluminal septal myocardial ablation (PTSMA). *(A)* Chest radiograph before PTSMA, showing severe cardiomegaly and pulmonary congestion. *(B)* TTE before PTSMA indicates stenosis of the left ventricular outflow tract (LVOT) in the systolic axial view (left panel) and peak LVOT pressure gradient of 119.7 mmHg in the apical four-chamber view (right panel). *(C)* Chest radiograph after PTSMA, showing a decreased cardiothoracic ratio. *(D)* TTE after PTSMA indicates stenosis at the LVOT in the systolic axial view (left panel) and a peak LVOT pressure gradient of 24.2 mmHg in the apical four-chamber view (right panel). LVOT PG, left ventricular outflow tract pressure gradient.

A 51-year-old woman with hypertension was transferred to our institution with cerebral haemorrhage and underwent immediate craniotomy for haematoma evacuation. The neurosurgical team initially observed cardiomegaly on chest radiography and attributed it to volume overload-induced heart failure and managed the patient with diuretics and vasodilators. However, the patient experienced multiple episodes of acute left heart failure, resulting in hypoxaemia-induced cardiac arrest that necessitated repeated cardiopulmonary resuscitation. The team consulted with the cardiology department, and the patient remained intubated. Electrocardiography revealed massive concentric hypertrophy. Chest radiography revealed severe cardiomegaly and pulmonary congestion (*Figure 1A*). Transthoracic echocardiography revealed a peak LVOT pressure gradient of 119.7 mmHg (*Figure 1B*). Laboratory data were as follows: serum brain natriuretic peptide, 1289 pg/ml (reference range: <18.4 pg/mlL); urea nitrogen, 56.4 mg/dL (reference range: 8.0–23.0 mg/dL); and creatinine, 1.15 mg/dL (reference range: 0.46–0.80 mg/dL). These findings indicated that excessive preload reduction with underlying HOCM exacerbated the LVOT obstruction, which was the primary cause of heart failure. Consequently, diuretics and vasodilators were discontinued, and fluid resuscitation was initiated. Thereafter, B-type natriuretic peptide levels decreased, and the patient was initially extubated. However, the patient developed acute pulmonary congestion again due to increased preload and after load-mismatch, necessitating reintubation. An increased preload volume can lead to heart failure due to elevated afterload, whereas a decreased preload volume can exacerbate LVOT obstruction. Under intubated management, volume control was re-attempted, but maintaining a preload worsened oxygenation, and renal failure gradually progressed, necessitating continuous haemodiafiltration (CHDF). Therefore, interventions targeting HOCM were required. Consequently, a calcium channel blocker (80 mg verapamil), beta-blocker (5 mg bisoprolol), and cibenzoline succinate (100 mg) were added intermittently, expecting a negative inotropic effect that would reduce the LVOT gradient; however, no improvement in LVOT obstruction was achieved. Of note, myosin inhibitors had not been approved in Japan during that time. Even during intubation, oxygenation rapidly deteriorated upon reduction of sedation, and the patient was unable to tolerate even minimal increases in afterload. Therefore, we determined that ‘direct’ intervention for LVOT obstruction was necessary. Septal reduction therapies (SRT) include surgical myectomy and PTSMA. Although the former was also considered, the acute clinical condition requiring MV and the high invasiveness associated with open-heart surgery led us to select PTSMA, which could be performed more promptly and less invasively.

**Figure 2:**
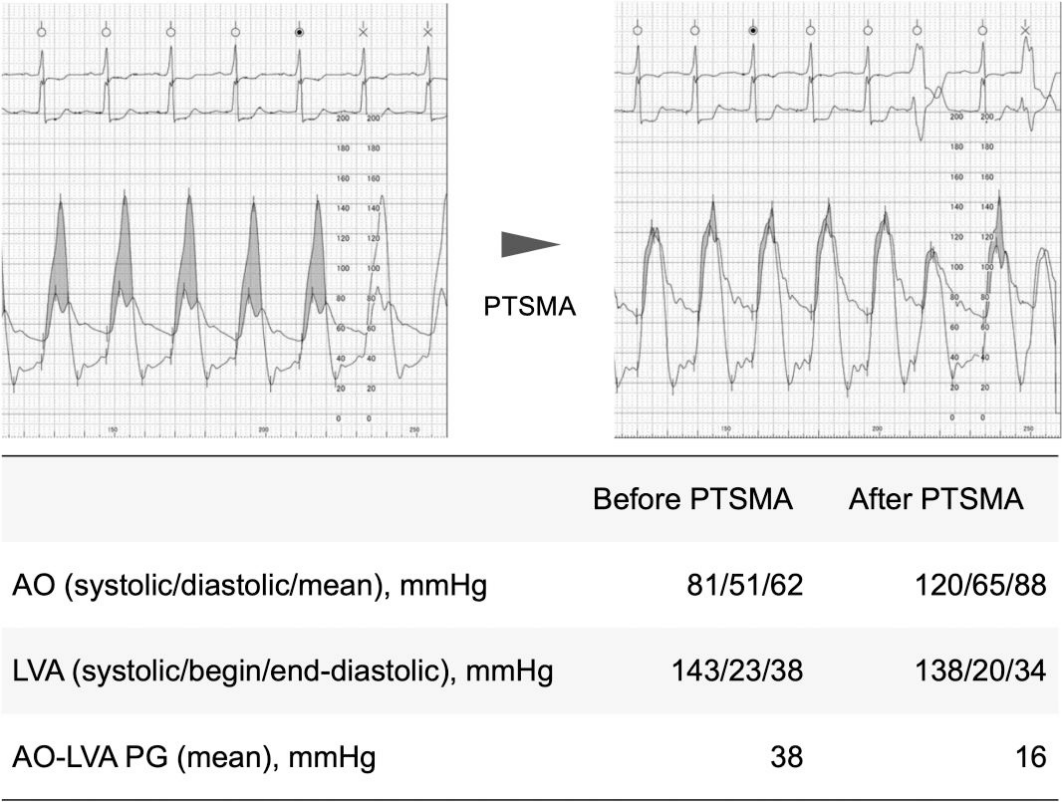
Pressure evaluation before and after percutaneous transluminal septal myocardial ablation (PTSMA). PTSMA decreases the aorta–left ventricle apex pressure gradient. AO, aorta; LVA, left ventricular apex; PG, pressure gradient; and PTSMA, percutaneous transluminal septal myocardial ablation.

**Figure 3:**
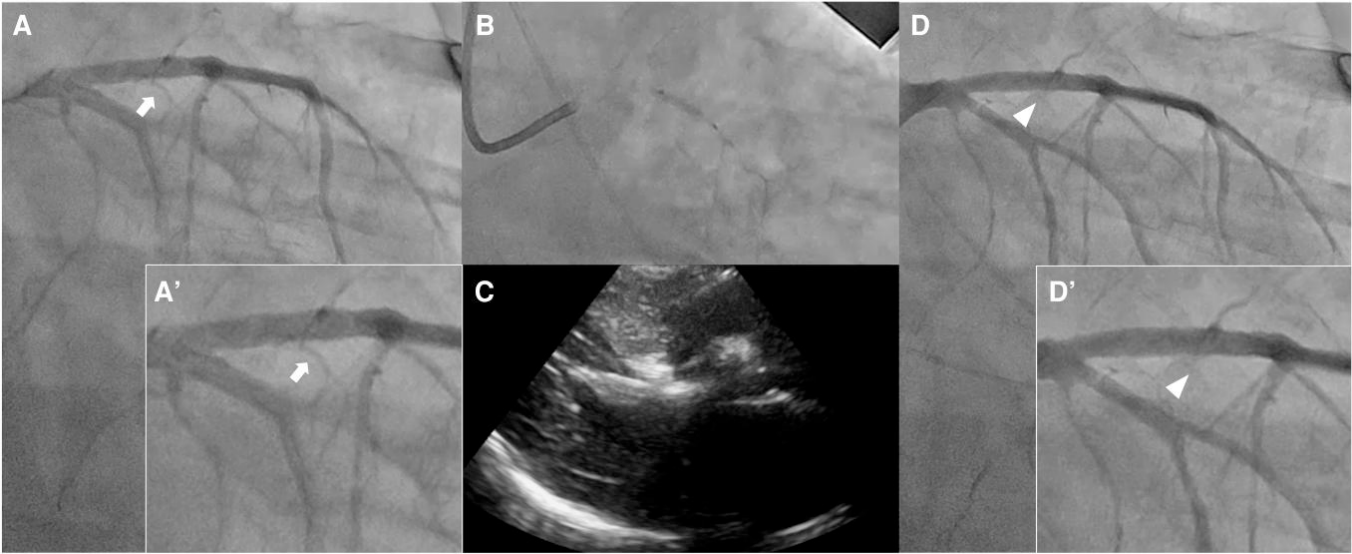
Percutaneous transluminal septal myocardial ablation (PTSMA) procedure. *(A)* First angiography of the left coronary artery with right anterior oblique-caudal view. White arrows indicate the target septal branches. *(A)* Magnified view of the same area. *(B)* Tip injection from the overwire balloon. *(C)* Septal myocardial opacification on echocardiography. *(D)* Final angiography of the left coronary artery with right anterior oblique-caudal view. White arrowheads indicate the disappearance of the target septal branch. d: magnified view of the same area.

Coronary angiography confirmed the absence of coronary artery stenosis. Pressure measurements were as follows: aorta, 102/64 mmHg; LV apex, 152/27 mmHg; mean pressure gradient, 38 mmHg; and peak pressure gradient, 62 mmHg (*Figure 2A*). After engagement with the MACH1 VL 3.5 guiding catheter (Boston Scientific, MA, USA), a branch directed towards the interventricular septum on the left ventricular side of the first major septal branch was selected using the SION guidewire (ASAHI Intech, Nagoya, Japan). Selective contrast injection confirmed septal myocardial opacification on echocardiography using an Emerge OTW 2.0 mm balloon (Boston Scientific, MA, USA) wedged in place. The vessel was ablated with 2 ml of absolute ethanol (*Figure 3*). After 20 min, the pressure gradient improved significantly (aorta, 138/20 mmHg; LV apex, 138/35 mmHg; mean pressure gradient, 4 mmHg; and peak pressure gradient, 12 mmHg), and the procedure was terminated (*Figure 2B*).

After PTSMA, aggressive volume reduction was performed using CHDF, and blood pressure was maintained without inducing LVOT obstruction, allowing the patient to reach a sufficiently dry state. The patient was successfully extubated one week after the procedure. Chest radiography revealed no pulmonary congestion (*Figure 1C*). Echocardiography also revealed a peak LVOT pressure gradient of 24.2 mmHg without evidence of aortic valve mid-systolic semiclosure or mitral valve systolic anterior movement (*Figure 1D*). Finally, the patient was transferred to a rehabilitation hospital to undergo maintenance dialysis and demonstrated no further episodes of heart failure approximately 10 months later with continued oral calcium channel blocker, beta-blocker, and cibenzoline succinate.

## Discussion

Positive-pressure ventilation can exacerbate LVOT obstruction in mechanically ventilated patients with HOCM by reducing venous return. A decreased preload reduces systemic blood pressure, whereas excessive fluid resuscitation may lead to pulmonary congestion, making extubation difficult, thereby making weaning from MV challenging.^5^

Conventional pharmacological approaches aim to attenuate LVOT obstruction by reducing contractility and increasing ventricular filling time.^6^ However, in patients with acute heart failure requiring MV, these therapies may be insufficient or even deleterious owing to their negative inotropic effects. Surgical myectomy remains the definitive treatment for severe LVOT obstruction, but is often not feasible in unstable, critically ill patients due to the high perioperative risk and need for specialized surgical expertise. Contrastingly, PTSMA is minimally invasive and can be performed promptly in a catheterization laboratory, rapidly reducing LVOT obstruction and allowing aggressive volume reduction. While PTSMA carries risks, including conduction disturbances requiring pacemaker implantation,^7^ they must be weighed against the complications of prolonged MV and inotropic support.

Although PTSMA is well-established for symptomatic relief in chronic HOCM, its role in the acute phase remains unclear. One case report described its use in patients with refractory heart failure due to exacerbated LVOT obstruction after surgical aortic valvular replacement.^4^ This case highlights the need for increased awareness of HOCM as a potential cause of extubation failure in critically ill patients. Given the increasing availability of catheter-based therapies, early recognition and intervention with PTSMA should be considered for mechanically ventilated patients with HOCM. Future studies to establish the long-term outcomes, patient selection, optimal timing, and procedural safety in critically ill patients are warranted.

## Conclusions

This case underscores the therapeutic potential of PTSMA as an acute intervention in critically ill patients with HOCM and haemodynamic instability. By alleviating LVOT obstruction, PTSMA may facilitate ventilator weaning and enable more effective preload management in medically refractory cases. Future studies should examine whether catheter-based septal reduction strategies could be considered in the acute management of obstructive physiology.

## Data Availability

Data underlying this article will be shared upon reasonable request from the corresponding author.

## Lead author biography

Dr Keisuke Nakabayashi graduated from the School of Medicine, Gunma University in 2008 and earned his PhD from Tokyo Women’s Medical University in 2017. He joined the Department of Cardiology at Kasukabe Chuo General Hospital in 2016, where he currently serves as Head of Cardiology. His main interests are interventional cardiology, complex coronary intervention, and acute cardiovascular care. He is dedicated to improving patient outcomes through advanced catheter-based therapies and multidisciplinary cardiovascular management. Outside of medicine, he enjoys spending time with his dog, Menma.
